# Equine Asthma in a Comparative Perspective: Cardiovascular and Neurological Manifestations of Asthma Across Different Species

**DOI:** 10.3390/ani15162371

**Published:** 2025-08-12

**Authors:** Dorota Dlugopolska, Natalia Siwinska, Agnieszka Noszczyk-Nowak

**Affiliations:** Department of Internal Medicine and Clinic of Diseases of Horses, Dogs and Cats, Faculty of Veterinary Medicine, Wrocław University of Environmental and Life Sciences, Grunwaldzki sq 47, 50-366 Wroclaw, Poland; dorota.dlugopolska@upwr.edu.pl (D.D.); agnieszka.noszczyk-nowak@upwr.edu.pl (A.N.-N.)

**Keywords:** asthma, cardiovascular system, neurological system, heart rate variability, renin-angiotensin-aldosterone, equine internal medicine

## Abstract

Asthma is a multifactorial respiratory disease that naturally occurs in horses, humans, and cats, presenting common clinical signs and species-specific mechanisms. This review addresses the impact of asthma on the cardiovascular and neurological systems, with a primary focus on horses. It highlights the need for new biomarkers beyond the respiratory system due to diagnostic difficulties in animals. A comprehensive literature search was conducted using PubMed and Google Scholar, focusing on cardiovascular and neurological manifestations of asthma in humans, horses, cats, and experimental animal models. Studies were qualitatively compared, noting species-specific differences and mechanisms. Humans with asthma show an increased risk of cardiovascular disease and elevated cardiac biomarkers during exacerbations, while horses develop pulmonary hypertension and vascular remodeling. Cats exhibit significant pulmonary vascular changes. Heart rate variability analysis reveals altered autonomic function in humans and horses. Increased peripheral airway innervation and cough reflex sensitivity are noted across species. The renin–angiotensin–aldosterone system (RAAS) plays a crucial role in asthma pathophysiology in murine models. Asthma impacts the cardiovascular and nervous systems differently across species, emphasizing the importance of comparative medicine. Future research should integrate cardiovascular, autonomic, and inflammatory pathways to develop effective therapeutic approaches in human and veterinary medicine, leveraging insights from naturally occurring asthma models.

## Simple Summary

Asthma is a complex disease that not only affects the respiratory tract but also has systemic consequences. This review explores how asthma impacts the cardiovascular and neurological systems across different species, with a primary focus on horses, and discusses potential biomarkers beyond the respiratory system. Asthma may contribute to pulmonary hypertension, structural changes in the heart, and alterations in vascular function. Changes in heart rate variability can serve as a potential non-invasive indicator of autonomic imbalance. Additionally, increased airway nerve density and enhanced cough reflex sensitivity have been observed in affected individuals. A hormonal pathway called the renin–angiotensin–aldosterone system (RAAS) appears to play a role in the inflammatory processes and airway remodeling associated with asthma. A comparative evaluation of different species may help identify new diagnostic markers and improve therapeutic approaches in both human and veterinary medicine.

## 1. Introduction

Asthma is a multifactorial respiratory disease that occurs naturally in humans, horses, and cats, exhibiting both common clinical signs and species-specific pathophysiological mechanisms. This review primarily evaluates the cardiovascular and neurological manifestations of asthma in horses, alongside relevant insights from human, feline, and experimental animal models. Given the challenges in precisely determining the severity of asthma in animals, coupled with existing gaps in our understanding of the disease, there is a need to identify novel asthma biomarkers, including those not directly related to the respiratory system. The aim of this review is to demonstrate how asthma affects the cardiovascular and nervous systems, to propose potential biomarkers for their assessment, and to highlight existing knowledge gaps along with future research directions.

## 2. Materials and Methods

A narrative literature review was conducted using the PubMed and Google Scholar databases. Search terms included combinations of the following keywords: “equine asthma”, “cardiovascular changes and asthma “, “feline asthma”, “vascular remodeling and asthma”, “neurological changes”, “human asthma”, “asthma and autonomic nervous system”, “asthma and heart rate variability”, and “renin-angiotensin-aldosterone system and asthma”. Each combination was searched individually. Articles were screened by title and abstract, and those that met the relevance criteria were read in full.

The following articles were included:

- Peer-reviewed original research articles;
- Review papers;
- Experimental studies on animal models;
- Studies comparing asthma between species;
- English-language articles.

The following articles were excluded:

- Abstract-only publications;
- Non-English studies;
- Case reports without mechanistic discussions.

A total of 90 articles were ultimately selected and referenced in this review. Key findings were categorized into thematic areas: cardiovascular manifestations, neurological dysfunction, heart rate variability, and the role of the renin–angiotensin–aldosterone system. The studies were compared qualitatively with attention to species-specific differences, mechanisms of action, and relevance for translational medicine.

## 3. Cross-Species Perspectives on Asthma: From Human Medicine to Veterinary Applications

Asthma only develops spontaneously under natural conditions in humans, cats, and horses. In other animal species, spontaneous asthma does not occur; therefore, for research and modeling purposes, the disease must be experimentally induced using standardized protocols [1].

Equine Asthma Syndrome (EA) is a complex lower respiratory tract disease characterized by variable symptoms, diagnosis, and treatment. In 2016, ACVIM introduced the term Equine Asthma Syndrome, dividing it into mild–moderate and severe asthma. Severe EA (sEA) results from hypersensitivity to airborne allergens, with complex immunological mechanisms and genetic predisposition influencing the immune response to organic dust, fungal spores, endotoxins, and grass pollen. The primary mechanism involves a delayed T-helper cell response, enhancing neutrophil recruitment, similar to late-onset asthma in humans. Neutrophils, IL-8, and IL-17 contribute to chronic airway inflammation; however, the cytokine profile remains unclear, suggesting mixed Th1, Th2, and Th17 responses and immunological heterogeneity [2]. Mild to moderate EA (mEA) also exhibits heterogeneous airway inflammation and inconsistent immunological pathways, suggesting that multiple etiological factors induce similar clinical presentations through distinct mechanisms [3].

Human asthma is a heterogeneous disorder characterized by airway hyperresponsiveness, cough, wheezing, and chronic inflammation. It presents with episodic respiratory symptoms and reversible airflow limitation [4]. Asthma is classified based on onset (childhood/late), type (allergic/non-allergic), and endotype (T2-high/low) [5]. Allergic asthma, which is common in childhood, exhibits a T2-high response with activation of IL-4, IL-5, IL-9, or IL-13, accompanied by eosinophilic inflammation [6]. T2-low asthma, often non-allergic and late-onset, is characterized by neutrophilic or paucigranulocytic inflammation, milder disease, a poor glucocorticoid response, and limited efficacy of T2-targeted therapies [7].

Feline asthma, which affects 1–5% of cats, is a chronic allergic respiratory disease characterized by airway inflammation, hyperresponsiveness, and airflow limitation. Manifestations include coughing, increased respiratory effort, and tachypnea [8]. Studies have shown that the fundamental immunological mechanism of feline asthma involves a Th2-type response and IgE production, which is analogous to the process occurring in humans [9]. Diagnostic tools involve radiography, CT scans, bronchoscopy, and bronchoalveolar lavage. Management relies on glucocorticoids and bronchodilators, similar to those used in human and equine asthma treatment [10].

In horses, the development of asthma results from the interplay of genetic predisposition and environmental factors, including dust, pollen, mold, mites, or bacterial endotoxins [3,11,12,13,14]. In human asthma, while environmental factors remain significant, the etiology is further complicated by contributions from obesity, dietary patterns, prior viral infections, and childhood medication use [5,15,16,17,18]. In cats, asthma exacerbations can be triggered by a variety of factors, including inhaled irritants, such as aerosols, certain medications, environmental allergens, cold air, and stress [10,19,20]. Therefore, for experimental research, asthma in other species, such as dogs, must be induced. The most common method involves sensitization with allergens, for instance, administering *Ascaris suum* larvae to provoke an allergic response [1,21]. Although dogs are susceptible to spontaneous allergic diseases, these most often present as atopic dermatitis rather than respiratory symptoms [21,22]. While some authors have mentioned the possibility of naturally occurring asthma in dogs, there is currently no clear evidence confirming its existence [23,24].

In mice, an allergic asthma model is usually established by repeatedly sensitizing animals with a specific allergen, most commonly ovalbumin (OVA, an egg white protein) or house dust mite extracts. The protocol typically involves intraperitoneal or subcutaneous administration of the allergen, together with an adjuvant, followed by airway challenge through inhalational exposure to the same allergen. This results in the development of key features characteristic of asthma, such as airway hyperresponsiveness, the infiltration of inflammatory cells, and airway remodeling [1,25]. These murine models are widely used for investigating asthma mechanisms and testing novel therapies [26,27].

Asthma in humans, horses, and cats is a complex disease characterized by diverse underlying mechanisms, yet it presents with similar clinical symptoms across all three species. A comparison of asthma across these three species is presented in Table 1. While these shared manifestations, such as airway hyperresponsiveness, chronic inflammation, and respiratory distress, are evident, each species exhibits distinct immunological pathways and disease triggers. Therefore, recognizing both the common clinical presentation and the mechanistic diversity among species is crucial; it deepens our understanding of asthma and highlights the importance of a comparative approach for advancing targeted diagnostics and treatment strategies.

## 4. Cardiovascular Implications of Asthma

### 4.1. Structural and Functional Consequences in the Cardiovascular System During Asthma

In horses, the impact of asthma on cardiac parameters has been investigated. Experimental studies, in which asthma episodes were induced, demonstrated that pulmonary obstruction associated with EA leads to the development of pulmonary hypertension. This, in turn, results in both structural and functional alterations of the right ventricle. When compared to control groups, horses with sEA in remission exhibited significant differences in several echocardiographic parameters, including increased right ventricular wall thickness, changes in the left ventricular end-systolic eccentricity index at the chordal level, and alterations in longitudinal myocardial strain [50].

Human asthma is associated with an increased risk of cardiovascular disease (CVD), and evidence suggests that both conditions may share common pathological mechanisms [5,17,45]. Inflammation, a key feature of asthma, appears to contribute to cardiovascular dysfunction. Elevated levels of mast cells, eosinophils, pro-inflammatory cytokines, and immunoglobulin E (IgE) have been documented not only in the lungs but also in the cardiac tissue and vasculature of individuals with asthma [51,52]. Asthma affects the cardiovascular system through cardiopulmonary interactions, where dynamic hyperinflation and increased respiratory effort alter cardiac function and output [53]. Key mechanisms include increased venous return, elevated right ventricular afterload, and ventricular interdependence [45]. The data indicate that diagnosed asthma is associated with a 38% increased risk of atrial fibrillation (AF). Moreover, there is a clear dose–response relationship, indicating that AF risk increases progressively with worsening asthma control. Uncontrolled asthma showed the highest risk increase (74% increased risk, hazard ratio 1.74), while partly controlled asthma demonstrated a 40% increased risk, and well-controlled asthma showed no statistically significant increase in AF risk [54]. Acute cardiac complications, such as arrhythmias, anginal chest pain, and myocardial infarction, have also been observed in patients with severe, refractory obstructive pulmonary disease, notably in those with status asthmaticus [16,45].

### 4.2. Cardiac Biomarkers in Asthma

In humans, increased levels of cardiac biomarkers such as troponin I (TnI), B-type natriuretic peptide (BNP), and creatine kinase-MB (CK-MB) have been observed during asthma exacerbations [55,56]. These elevations are considered clinically relevant and have been associated with myocardial dysfunction in patients experiencing acute manifestations of the disease [57]. Similarly, increased concentrations of NT-proBNP and troponin T (TnT) have been reported in studies evaluating exacerbations of chronic obstructive pulmonary disease (COPD) in humans [58]. In contrast, studies in horses have not demonstrated significant changes in the levels of cardiac biomarkers, including TnI, TnT, and CK, during asthma episodes [50].

### 4.3. Vascular Remodeling

One study reported that asthmatic horses demonstrated significant thickening of the walls of the pulmonary arteries, with a 7% increase in surface area observed in vivo and a 12% increase post-mortem compared to control animals. This thickening was attributed to an increase in pulmonary arterial smooth muscle mass (PASM), while the extracellular matrix (ECM) remained unchanged. Notably, vascular remodeling was found to be fully reversible only after 12 months of antigen avoidance. No improvement was observed following short-term antigen avoidance (2–4 months) or with the use of inhaled corticosteroids combined with six months of antigen avoidance. These changes were most prominent in the apical and caudodorsal regions of the lung [11]. The observed increase in PASM confirms the presence of vascular remodeling in asthmatic horses, mirroring similar findings described in human COPD [59]. The hypertrophy of PASM may be explained by chronic hypoxia-induced vasoconstriction, as observed in other animal models [11].

Based on the available research, vascular changes in feline asthma demonstrate significant alterations in pulmonary vasculature [49]. In the airways of asthmatic cats, several notable vascular changes have been documented. The bronchial submucosa demonstrates an increased number of small blood vessels and enhanced angiogenesis, resulting in the development of an extensive vascular network, as confirmed by immunohistochemical analysis using the CD31 marker. Within the pulmonary arteries, there is marked hyperplasia of the tunica media and hypertrophy of the vascular smooth muscle layer. These changes are mediated by multiple molecular mechanisms, including a key role for transforming growth factor beta 1 (TGFβ-1), which promotes the proliferation of vascular smooth muscle cells. Angiogenesis is further regulated by various cytokines and mediators produced by epithelial and mesenchymal cells. These vascular alterations may have important clinical consequences, including increased vascular resistance, the development of pulmonary hypertension, and secondary changes in the myocardium, such as interstitial fibrosis. Vascular changes represent a critical aspect of airway remodeling in cats with asthma and may influence disease progression and its complications. These findings parallel similar pathological alterations seen in both human and equine asthma, thus underscoring shared pathophysiological mechanisms across species and highlighting the value of feline models in advancing our understanding of vascular remodeling in asthma [60].

Asthma induces notable cardiovascular changes in humans, horses, and cats, but the nature of these alterations varies by species. In humans, asthma is linked to increased cardiovascular risk and biomarker elevation during exacerbations. Horses mainly develop pulmonary hypertension and vascular remodeling, while cats show pronounced changes in pulmonary vessels. Understanding these interspecies differences is important for improving diagnostic and therapeutic approaches.

## 5. Heart Rate Variability as a Non-Invasive Marker of Autonomic Dysfunction in Asthma

Heart rate variability (HRV) refers to fluctuations in the time intervals between consecutive heartbeats [61]. It serves as a non-invasive measure of autonomic nervous system (ANS) regulation, reflecting the dynamic balance between sympathetic nervous system (SNS) and parasympathetic nervous system (PNS) influences. Proper equilibrium between SNS and PNS activity is essential for maintaining physiological homeostasis. Disturbances in autonomic function can be assessed through time domain, frequency domain, and non-linear analyses of HRV [62].

In the time domain, commonly used parameters include the standard deviation of NN intervals (SDNN), square root of the mean of the sum of the squares of differences between successive NN intervals (RMSSD), and percentage of successive NN intervals differing by more than 50 ms (PNN50). Frequency domain analysis includes low-frequency (LF, 0.04–0.15 Hz), high-frequency (HF, 0.15–0.4 Hz), and the LF/HF ratio. Additionally, non-linear HRV indices provide insight into the complexity of ANS regulation and include parameters such as the Poincaré plot standard deviation perpendicular to the line of identity (SD1), the Poincaré plot standard deviation along the line of identity (SD2), and the SD1/SD2 ratio [63]. HRV is primarily derived from ECG recordings and serves as a valuable tool in assessing cardiovascular function and autonomic regulation [64].

Studies conducted in children with stable, chronic asthma have demonstrated the presence of autonomic dysfunction in cardiac regulation. These abnormalities are most likely attributable to increased parasympathetic activity, as evidenced by short-term frequency domain HRV analysis. When compared to healthy controls, children with asthma show significantly higher high-frequency (HF) values, lower low-frequency (LF) values, and a reduced LF/HF ratio [18]. A systematic review and meta-analysis on HRV in children and adolescents with asthma confirmed a reduction in global HRV and sympathetic modulation. These findings suggest that HRV may serve as a non-invasive biomarker of autonomic dysfunction in asthma. However, its prognostic value and association with disease severity require further investigation and standardization of measurement protocols [65].

Given the parallels between human and equine asthma, research has investigated HRV alterations in horses with Equine Asthma Syndrome. The findings indicate that autonomic nervous system control is reduced in affected horses, with a relative increase in parasympathetic heart modulation [66]. These observations suggest that HRV may serve as a valuable non-invasive tool for assessing autonomic dysfunction in equine asthma.

HRV analysis provides valuable insights into autonomic dysfunction in asthma, with potential applications in both human and veterinary medicine. Future research should focus on standardizing HRV measurement protocols and exploring its clinical utility as a diagnostic and prognostic biomarker in asthma management.

## 6. The Role of the Renin–Angiotensin–Aldosterone System in the Pathophysiology of Asthma

### 6.1. General Overview of the Renin–Angiotensin–Aldosterone System

When evaluating asthma in the context of cardiology, it is essential to consider the role of the renin–angiotensin–aldosterone system (RAAS), which serves as a primary regulator of cardiovascular and fluid–electrolyte homeostasis [67]. Since RAAS operates systemically, any disturbances within its cascade can lead to dysfunction in multiple organs and contribute to the development of various diseases, including neurodegenerative and cardiovascular disorders [68]. Angiotensin II (AngII) is a principal effector of the RAAS and is primarily responsible for inducing vasoconstriction and increasing sodium reabsorption in the nephrons, which results in elevated blood pressure and increased circulating blood volume [69]. Under physiological conditions, this mechanism regulates renin secretion through a negative feedback loop [70]. However, beyond its hemodynamic effects, AngII also exerts pro-inflammatory properties by promoting the recruitment of immune cells and activating the NF-κB pathway in monocytes, macrophages, endothelial cells, and vascular smooth muscle cells [71]. This activation leads to the synthesis of proinflammatory mediators, further amplifying the inflammatory response. Excessive RAAS activation has been associated with chronic inflammation, increased fibrosis, and excessive cellular proliferation, contributing to disease progression [72]. Renin secretion plays a crucial role in regulating RAAS activity. It is controlled through four primary mechanisms: negative feedback inhibition by AngII acting on juxtaglomerular cells, the detection of chloride ion concentration fluctuations, baroreceptor activation, and the stimulation of the sympathetic nervous system [73]. Sympathetic activation increases renin secretion through the release of noradrenaline from sympathetic nerve endings, which acts on beta1-receptors in the juxtaglomerular apparatus (JGA), as well as through calcium channel blockers, which activate baroreceptors [74].

### 6.2. The Role of the Renin–Angiotensin System (RAS) in Experimental Murine Models of Asthma

**Figure 1:**
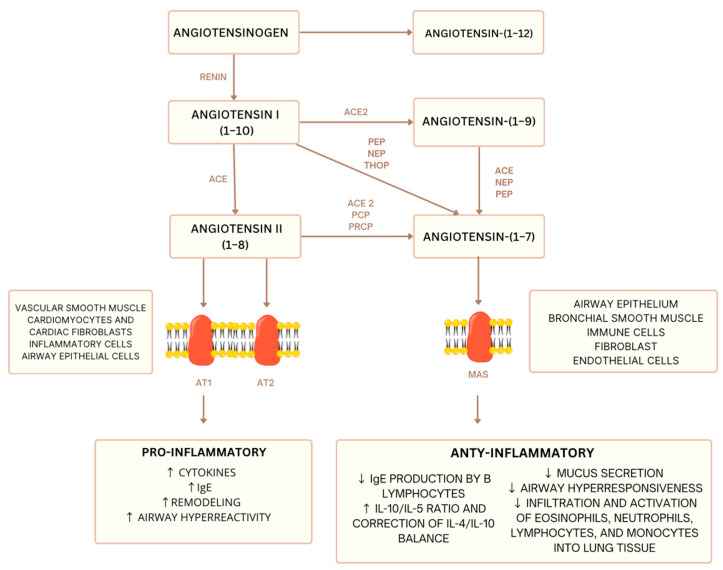
Simplified diagram of the renin–angiotensin system (RAS): balance between pro- and anti-inflammatory pathways [67,68]. ACE—Angiotensin-Converting Enzyme, ACE2—Angiotensin-Converting Enzyme 2, PEP—Prolyl Endopeptidase, NEP—Neutral Endopeptidase (Neprilysin), THOP—Thimet Oligopeptidase, PCP—Prolyl Carboxypeptidase, PRCP—Prolylcarboxypeptidase, AT1—Angiotensin II Type 1 Receptor, AT2—Angiotensin II Type 2 Receptor, MAS—Mas Receptor. downward arrow—decrease in level, upward arrow—increase in level.

Experimental and clinical studies have demonstrated that the renin–angiotensin system (RAS) is a key modulator involved in the pathophysiology of asthma in a murine model [75]. Figure 1 provides a simplified representation of the inflammatory balance regulated by RAS. The RAS, primarily known for its role in blood pressure regulation, significantly influences inflammatory processes and tissue remodeling, making it central to the development and progression of asthma [76]. Notably, the detrimental effects of the AngII/AT1 pathway have been confirmed in inflammation, airway remodeling, and airway hyperresponsiveness in asthma [75].

Angiotensin II (AngII), the primary effector peptide of the RAS, acting through its type 1 receptor (AT1), promotes inflammation by stimulating the release of proinflammatory cytokines, such as IL-6 and TNF-α, from airway epithelial and immune cells. Airway remodeling, characterized by structural changes like increased smooth muscle mass and collagen deposition, is exacerbated by AngII/AT1 signaling, contributing to irreversible airflow obstruction. Airway hyperresponsiveness (AHR), a hallmark of asthma, is heightened by AngII’s ability to sensitize airways to stimuli [75].

Elevated levels of AngII and renin have been reported in patients with severe, acute asthma, with higher plasma concentrations observed during exacerbations compared to healthy controls [76,77].

Furthermore, AngII enhances bronchoconstriction induced by methacholine and endothelin-1. Methacholine, a muscarinic receptor agonist, is commonly used to assess AHR, and AngII potentiates its bronchoconstrictive effects [76]. Similarly, endothelin-1, a potent vasoconstrictor and bronchoconstrictor peptide, synergistically interacts with AngII to amplify airway narrowing [78].

AngII induces the contraction of human airway smooth muscle cells by activating the RhoA/ROCK2 signaling pathway, which increases calcium sensitization and enhances contractile force. This mechanism contributes to bronchospasm, as confirmed by in vitro studies showing that RhoA/ROCK2 inhibition attenuates AngII-induced contraction [79].

Research in rats has demonstrated a significant role of AngII in asthma pathophysiology. While renin is absent in rat airways, the presence of AT1A, AT1B, and AT2 receptors, along with angiotensinogen and ACE1, suggests a local renin–angiotensin system dependent on external renin supply. Although AngII causes modest direct bronchial smooth muscle contraction through AT1 receptors in rat models, it significantly enhances responsiveness to various contractile agents via p42/44 ERK pathway activation [76].

The clinical relevance of these findings is supported by observations in humans, which show elevated plasma renin and AngII levels during severe asthma attacks and AngII-induced bronchoconstriction in mild asthma patients. These rat studies suggest potential therapeutic approaches through AngII pathway modulation.

Excessive AngII production within the RAS plays a key role in allergic pulmonary disease pathogenesis. However, studies have identified a counter-regulatory pathway, the Ang-(1–7)/Mas receptor axis, which exerts protective effects against the AngII/AT1 pathway. Research has demonstrated that Ang-(1–7) or AVE 0991 (a non-peptide of Ang-(1–7)) exhibits anti-inflammatory properties and prevents pathological tissue remodeling in various disease states. Additionally, it possesses antifibrotic effects in both short-term and long-term models of allergic airway inflammation [76].

An increasing body of evidence suggests the therapeutic potential of the Ang-(1–7)/Mas receptor axis in chronic asthma, although further studies are required to fully elucidate its mechanisms and assess its clinical efficacy. To the best of the authors’ knowledge, the RAAS has not yet been studied in equine asthma. Based on data from studies in other animal species and humans, similar research in horses could contribute to a better understanding of this disease and further advancement in this field, particularly given the potential for developing new therapeutic approaches [80].

## 7. Neuroregulatory Changes in Asthma

Inflammatory response and airway hyperresponsiveness are closely interdependent due to bidirectional interaction between the nervous and immune systems. Airway inflammation affects neuronal activity, including sensory nerve function and synaptic transmission. Inflammation increases neurotrophin concentration in bronchoalveolar lavage fluid in asthma patients and animal models [72]. Sensory airway nerves enhance immune response via the release of neuropeptides and neurotransmitters, resulting in neurogenic inflammation. This neuroimmune interaction amplifies airway inflammation and hyperresponsiveness in allergic asthma. Neurotrophins increase neuropeptide content in sensory nerves, affecting the survival/activation of inflammatory cells and airway hyperresponsiveness [81]. Continuously produced during allergic inflammation, neurotrophins act as long-term modulators, amplifying inflammatory signals [82]. Sensory nerve dysfunction in asthma can lead to irritative cough and airway hyperresponsiveness [83]. This is more evident in eosinophilic inflammation, where increased nerve density amplifies sensitivity to triggers. Cough begins when receptors in the trachea, carina, airway bifurcations, and distal bronchi detect stimuli. These receptors also exist in the sinuses, auditory canals, pleura, and stomach. Once activated, sensory signals travel via the vagus nerve to the medullary cough centre. This centre coordinates a response through the vagus, phrenic, and spinal motor nerves. The cough reflex occurs in three stages. The inspiratory phase increases lung volume with deep inhalation. During the compression phase, the larynx closes while chest muscles contract, raising intrathoracic pressure. Finally, during the expiratory phase, the glottis opens, allowing for high airflow to expel mucus [84]. This reflex becomes exaggerated in asthma, contributing to persistent cough and airway irritation.

Research from 2023 demonstrated increased innervation of the peripheral airways in horses with severe asthma, potentially contributing to the remodelling of the airway smooth muscle and exacerbating the severity of the disease. Dysfunction in bronchial innervation may play a role in the onset and persistence of severe equine asthma [85].

The respiratory tract’s afferent and efferent nerves travel through the vagus nerve, influencing airway smooth muscle tone and breathing patterns [86]. Additionally, they impact vascular tone, mucus secretion, and inflammation by interacting with muscarinic receptors on various cell types [87]. In horses, airflow obstruction during asthma exacerbations is mainly driven by bronchoconstriction and airway hyperresponsiveness (AHR) in response to inhaled environmental antigens [88]. This condition is supported by bronchodilator treatment, which often results in a 60% to 70% improvement in lung function [89].

Based on the current literature, there is no evidence describing morphological or functional changes in the nervous system of cats with asthma. The available studies and reviews primarily address immunological mechanisms, inflammatory changes, and structural remodeling of the airways, without mentioning neurological alterations or symptoms [48]. Therefore, further research is needed to determine whether feline asthma has any impact on the nervous system.

Asthma is associated with altered airway innervation and sensory nerve dysfunction, leading to stronger responses to stimuli. Increased nerve density, particularly in eosinophilic inflammation, amplifies cough reflex sensitivity and airway hyperresponsiveness. In horses with severe asthma, increased airway innervation may contribute to airway remodeling and disease progression. The cross-species neurological and cardiovascular consequences of asthma are summarized in Table 2.

## 8. Summary

Asthma represents a complex multisystem disorder that extends beyond its primary respiratory manifestations, significantly impacting both the cardiovascular and autonomic nervous systems. This comprehensive review focuses on comparative insights among human, equine, and feline asthma—the only three species in which the condition occurs spontaneously.

The comparison reveals both shared mechanisms and species-specific differences. In cardiovascular aspects, humans show elevated cardiac biomarkers during exacerbations, while horses do not exhibit such changes. Horses demonstrate significant pulmonary artery wall thickening, while cats show distinct vascular remodeling, including increased bronchial submucosa vascularization and arterial muscle layer hypertrophy. The renin–angiotensin–aldosterone system (RAAS) plays a crucial role in asthma pathophysiology, particularly through angiotensin II’s effects on airway inflammation and remodeling. The counter-regulatory Ang-(1–7)/Mas receptor pathway shows promise as a therapeutic target, although the RAAS remains unstudied in equine asthma, despite its potential significance.

Heart rate variability (HRV) analysis has emerged as a valuable non-invasive tool for assessing autonomic dysfunction across species. Both humans and horses with asthma exhibit altered sympathovagal balance, although standardized measurement protocols are still needed for clinical application.

## 9. Conclusions

Understanding these interspecies similarities and differences is crucial for advancing diagnostic methods and treatment strategies. Future research should aim to validate cardiovascular and autonomic parameters, such as heart rate variability, as non-invasive biomarkers of asthma severity and control in animals. Studies in horses with naturally occurring asthma may provide valuable insights into the progression and systemic impact of the disease. In addition, experimental investigations into the role of the RAAS in equine asthma could clarify its contribution to airway remodeling and inflammation, potentially leading to the development of new therapeutic strategies.

This cross-species perspective highlights the value of comparative medicine in developing comprehensive treatment strategies for asthma, acknowledging both shared mechanisms and species-specific variations in disease manifestation and progression.

## Figures and Tables

**Table 1:** Comparison of asthma in humans, horses, and cats—general aspects.

Feature	Humans	Horses	Cats
Prevalence	334 million people worldwide, 1–18% of population in different countries [28,29]	10–20% of adult horses in temperate climate (sEA), 60% or more may have mild/moderate form [2,30,31,32]	1–5% of cat population [33]
Immunological mechanisms	T2-high (allergic): presence of eosinophils, IL-4, IL-5, IL-13, IgE; T2-low (non-allergic): presence of neutrophils, Th1/Th17 response; mixed cytokine profiles [5,15,34]	Heterogeneous immune response; neutrophil dominance in severe form; IL-4, IL-5 expression in BAL; increased expression of IL-1β, IL-8, IFNγ, TNFα, IL-17 [14,35,36,37,38,39,40]	Dominant T2-high response; eosinophilic airway inflammation; IgE-dependent activation; similar immunological profile to human allergic asthma; presence of Th2 cells [20,41,42,43]
Types/phenotypes	Allergic (early onset); non-allergic (late onset); obesity-related; aspirin-induced; eosinophilic; neutrophilic [5,17,44]	Mild/moderate (mEA) with various inflammation types; severe (sEA) with neutrophil dominance; different phenotypes depending on environmental factors [2,3,39]	Allergic form of asthma; chronic bronchitis as a possible equivalent of non-allergic asthma; dominance of allergic type [9,10,20,33]
Clinical symptoms	Dyspnea; wheezing; cough; chest tightness; reduced exercise tolerance; nocturnal attacks [5,16,45,46]	Cough; respiratory effort; exercise intolerance; nasal discharge; wheezing; exacerbations with dust/hay exposure [2,3,13,47]	Chronic cough; wheezing; exercise-induced dyspnea; episodic respiratory distress; reversible airway obstruction [10,33,48,49]

**Table 2:** Impact of asthma on the cardiovascular and nervous systems.

Location	Humans	Horses	Cats
Cardiovascular changes	Increased risk of cardiovascular disease, atrial fibrillation, cardiac arrhythmias, and right ventricular changes; elevated TnI, BNP, CK-MB during exacerbations [54,56,58]	Pulmonary artery wall thickening (7% larger area in vivo, 12% post-mortem); no increase in cardiac biomarkers (TnI, TnT, CK); structural and functional changes in right ventricle; pulmonary hypertension [11,12,50]	Increased vascularization in bronchial submucosa; vascular muscle layer hypertrophy; extensive vascular network confirmed by CD31 marker; changes in TGFβ-1 expression; development of pulmonary hypertension [60]
HRV changes	Reduced total heart rate variability; altered sympathetic–parasympathetic balance; elevated HF values; reduced LF values; decreased LF/HF ratio; disturbed autonomic modulation [18,63,65]	Reduced autonomic cardiac control; relative increase in parasympathetic modulation; changes in SDNN, RMSSD parameters; disturbed autonomic regulation [66]	No detailed HRV studies in cats with asthma; presumed autonomic control disturbances similar to humans
Neurological changes	Airway hyperresponsiveness; increased sensory nerve sensitivity; bronchial receptor reflex disturbances; increased nerve density in airways; parasympathetic system dysfunction [18,46,82,87]	Increased peripheral airway innervation; bronchial innervation dysfunction; airway hyperresponsiveness; increased number and surface area of peribronchial nerves; impact on smooth muscle remodeling [11,86,89]	Similarities in non-adrenergic, non-cholinergic control of airway diameter to humans; preserved response to beta-agonists; hyperresponsiveness to methacholine; reversibility of bronchospasm [33,90]

## Data Availability

No new data were collected or analyzed in this study. Data sharing is not applicable to this article.
